# Chemical Swarming: Depending on Concentration, an Amphiphilic Ruthenium Polypyridyl Complex Induces Cell Death via Two Different Mechanisms

**DOI:** 10.1002/chem.201600927

**Published:** 2016-07-04

**Authors:** Bianka Siewert, Vincent H. S. van Rixel, Eva J. van Rooden, Samantha L. Hopkins, Miriam J. B. Moester, Freek Ariese, Maxime A. Siegler, Sylvestre Bonnet

**Affiliations:** ^1^ Leiden Institute of ChemistryLeiden University2300 RALeidenNetherlands), FAX; ^2^ Department of Physics & AstronomyVrije Universiteit Amsterdam1081 HVAmsterdamNetherlands; ^3^ Small Molecule X-ray Crystallography FacilityJohns Hopkins UniversityBaltimoreMD21218USA

**Keywords:** bioinorganic chemistry, cell death, cytotoxicity, metallosomes, ruthenium

## Abstract

The crystal structure and in vitro cytotoxicity of the amphiphilic ruthenium complex [**3**](PF_6_)_2_ are reported. Complex [**3**](PF_6_)_2_ contains a Ru−S bond that is stable in the dark in cell‐growing medium, but is photosensitive. Upon blue‐light irradiation, complex [**3**](PF_6_)_2_ releases the cholesterol–thioether ligand **2** and an aqua ruthenium complex [**1**](PF_6_)_2_. Although ligand **2** and complex [**1**](PF_6_)_2_ are by themselves not cytotoxic, complex [**3**](PF_6_)_2_ was unexpectedly found to be as cytotoxic as cisplatin in the dark, that is, with micromolar effective concentrations (EC_50_), against six human cancer cell lines (A375, A431, A549, MCF‐7, MDA‐MB‐231, and U87MG). Blue‐light irradiation (*λ*=450 nm, 6.3 J cm^−2^) had little influence on the cytotoxicity of [**3**](PF_6_)_2_ after 6 h of incubation time, but it increased the cytotoxicity of the complex by a factor 2 after longer (24 h) incubation. Exploring the unexpected biological activity of [**3**](PF_6_)_2_ in the dark elucidated an as‐yet unknown bifaceted mode of action that depended on concentration, and thus, on the aggregation state of the compound. At low concentration, it acts as a monomer, inserts into the membrane, and can deliver [**1**]^2+^ inside the cell upon blue‐light activation. At higher concentrations (>3–5 μm), complex [**3**](PF_6_)_2_ forms supramolecular aggregates that induce non‐apoptotic cell death by permeabilizing cell membranes and extracting lipids and membrane proteins.

## Introduction

Collective behavior belongs to the most successful evolutionary models in nature; a single bee prick only kills the most sensitive victims, whereas an attack by swarming bees kills an Asian giant hornet by producing heat.^1^ Can this concept of biological swarming be applied to chemistry? Herein, we demonstrate how the chemical modification of a poorly toxic drug‐like compound with a nontoxic lipophilic ligand leads to a strong biological response mediated either by individual molecules or by their supramolecular assemblies.

The drug‐like structure of interest is a ruthenium–polypyridyl complex: [Ru(tpy)(bpy)(OH_2_)]^2+^ ([**1**]^2+^; tpy=2,2′;6′,2“‐terpyridine, bpy=2,2′‐bipyridine). For most metallodrugs, specific DNA and/or protein interactions have been proposed as the mode of action.^2^ In principle, aqua complex [**1**]^2+^ is a strong electrophile and its binding to DNA^3^ and proteins,^4^ which has been thoroughly investigated in the past, suggested that such compounds may be used as an anticancer agent.3a, 3b, 4 However, Reedijk et al. demonstrated that [Ru(tpy)(bpy)Cl]Cl, which in water hydrolyzes into [**1**]^2+^, is poorly cytotoxic.3b Probably, complex [**1**]^2+^ loses its ability to bind to biomolecules and become cytotoxic before it even enters the cell, by undergoing quick ligand‐exchange reactions with nucleophiles present in media (Figure S2 in the Supporting Information); thus forming an inactive complex. Exchanging the labile aqua or chloride ligand with a much more strongly bound ligand, L (e.g., a thioether‐, a nitrile‐, or pyridine‐based ligand), may prevent such undesired reactions in the dark. In addition, ruthenium complexes such as [Ru(tpy)(bpy)(L)]^2+^ are photochemically active because visible‐light irradiation leads to ligand‐exchange reactions that do not occur in the dark.^4^, ^5^ Such photosubstitution reactions have been proposed as a way to trigger the toxicity of anticancer metallodrugs with spatial and temporal resolution.5a, 5g, 5h, 6 Likewise, photosubstitution of the protecting monodentate ligand L in [Ru(tpy)(bpy)(L)]^2+^ may activate the complex by producing [**1**]^2+^ inside a cell.

**Figure 1:**
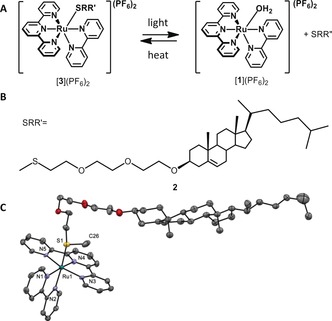
A) Formation of the active compound [**1**](PF_6_)_2_ through light expulsion of the thioether ligand [**3**](PF_6_)_2_. B) Chemical structure of the investigated thioether–cholesterol ligand **2**. C) Displacement ellipsoid plot (50 % probability level) of cationic [**3**]^+^, as observed in its crystal structure at 110(2) K. Only one of the two crystallographically independent molecules is shown. Counteranions, hydrogen atoms, and disorder have been omitted for clarity. Characteristic bond lengths [Å]: Ru1−N1=2.090(5), Ru1−N2=2.072(4), Ru1−N3=2.081(5), Ru1−N4=1.976(5), Ru1−N5=2.065(5), Ru1−S1=2.3639(14).

Herein, we report on reactivity and cytotoxicity studies with compound [**3**](PF_6_)_2_, which is a conjugate of [**1**](PF_6_)_2_ and the thioether–cholesterol ligand **2** (Figure 1). For metallodrugs, ligands can, in principle, be utilized to alter drug‐like parameters, for example, solubility and/or stability, or to introduce functional moieties, such as cancer‐cell targeting groups or linkers to a drug carrier. In [**3**](PF_6_)_2_, the cholesteryl group was initially proposed as a lipid bilayer anchor to deliver the complex to cancer cells by using liposomes.^7^ However, cholesterol is also lipophilic, which is expected to dramatically change the partition coefficient (log *P*)7b–7d and localization of [**3**]^2+^ compared with that of [**1**]^2+^. The biological properties of [**3**](PF_6_)_2_ were studied herein in the absence of any liposome drug‐delivery system. As shown below, the amphiphilic character of [**3**](PF_6_)_2_ resulted in an unexpectedly high and nonselective cytotoxicity profile against a range of human cancer cell lines. Chemical biological investigations and in vitro light irradiation experiments characterized a cell death mechanism that depended on concentration.

## Results and Discussion

Compound [**3**](PF_6_)_2_ was synthesized according to previous reports.7a Single crystals suitable for X‐ray structure determination were grown by vapor diffusion of diethyl ether into a solution of the compound in ethyl acetate. The structure confirmed the coordination of ligand **2** to ruthenium through the sulfur atom (Figure 1 C). The photochemical release of **2** by the blue‐light irradiation of [**3**]^2+^ (*λ*=455 nm, 10.5 mW cm^−2^) was studied in the absence of cells, but under the conditions used for in vitro toxicity experiments,^8^ that is, in Opti‐MEM complete cell‐growing medium (see composition in the Supporting Information). Within 8 min of irradiation (5.0 J cm^−2^), a bathochromic shift of the metal‐to‐ligand charge transfer (MLCT) absorption band of the complex was observed, from *λ*_max_=454 nm for [**3**]^2+^ to *λ*_max_=480 nm, which was characteristic for [**1**]^2+^ (Figure S3 in the Supporting Information). According to ESI‐MS results, the signal for [**3**]^2+^ at *m*/*z* 519.6 indeed disappeared and an intense signal at *m*/*z* 571.3 appeared for [**2**+Na]^+^ (calcd *m*/*z* 571.4; Figure S4 in the Supporting Information); this demonstrates that the photochemical release of ligand **2** (Figure 1 A) also occurs in the medium.

**Figure 2:**
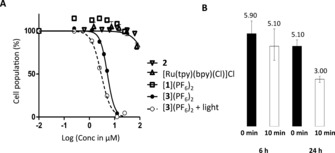
A) Logarithmic dose–response curve for A549 lung cancer cells after treatment for 24 h with **2** (inverted triangles), [Ru(tpy)(bpy)Cl]Cl (triangles), [**1**](PF_6_)_2_ (squares), and [**3**](PF_6_)_2_ (filled dots) in the dark. The empty dots data points and the dashed curve represent the dose–response curve for cells treated with [**3**](PF_6_)_2_ and irradiated with blue light (*λ*=455 nm, 10 min, 6.3 J cm^−2^). B) Effective concentrations (EC_50_ in μm with 95 % confidence interval) in the dark and after blue‐light irradiation (*λ*=450 nm, 10 min, 6.3 J cm^−2^) of [**3**](PF_6_)_2_ on A549 cancer cells after 6 and 24 h of incubation.

The cytotoxicity of [**1**](PF_6_)_2_, **2**, and [**3**](PF_6_)_2_ was investigated on six different human cancer cell lines derived from photodynamic therapy (PDT)‐relevant malignant tissues (skin, lung, brain, and breast; see the Experimental Section for more details). Briefly, 24 h after seeding, the cells were incubated with the compounds for 6 or 24 h, the media was refreshed, and blue‐light irradiation was performed (*λ*=455 nm, 10 min, 6.3 J cm^−2^). Following irradiation, the cells were incubated for an additional 48 h, and then counted by using the sulforhodamine B (SRB) assay (Figure 2 A).^9^ When possible, the effective concentration (EC_50_) leading to 50 % lower cell population compared to a drug‐free control was determined in µm (Table 1). In the dark, neither **2** nor the aqua complex [**1**](PF_6_)_2_ showed any cytotoxicity. Surprisingly, however, complex [**3**](PF_6_)_2_ was found very cytotoxic, that is, with micromolar EC_50_ values for all cancer cell lines tested, whereas cisplatin showed expected increased cytotoxicity against faster proliferating cells (e.g., A375) and decreased cytotoxicity against a slower proliferating cell line (e.g., MDA‐MB‐231).^10^ Meanwhile, the results of blue‐light photocytotoxicity studies (Figure 2 and Table S1 in the Supporting Information) were intriguing. After 6 h of incubation, the cytotoxicity was barely influenced by irradiation, whereas after 24 h of incubation the activity increased by a factor of two. Taken together, the unspecific toxicity of [**3**](PF_6_)_2_ in the dark suggested that this compound might not interfere with cell proliferation through DNA binding, whereas the time‐dependent, light‐enhanced cytotoxicity suggested an adenosine triphosphate (ATP)‐dependent internalization process.

**Table 1:** Cytotoxicity of [**1**](PF_6_)_2_, **2**, and [**3**](PF_6_)_2_ in the dark given as effective concentrations (EC_50_) in μm with a confidence interval of 95 %.

Cell line	EC_50_ [μm]			
	[**1**](PF_6_)_2_	**2**	[**3**](PF_6_)_2_	Cisplatin
A375	>150	>60	6.5±1.1	1.9±0.2
A431	>150	>60	6.2±0.9	3.6±0.6
A549	>150	>60	5.2±0.4	4.3±0.7
MCF‐7	>150	>60	5.0±1.1	2.6±0.4
MDA‐MB‐231	>150	>60	6.0±0.8	>25
U87Mg	>150	>60	6.5±2.4	5.9±1.0

MALDI‐MS was used to obtain qualitative information about drug uptake. An immediate advantage of MALDI‐MS compared with the more usual inductively coupled plasma (ICP) MS technique is the ease of sample preparation.^11^ At several time points of drug incubation (1, 6, and 24 h), MS spectra were measured directly from thoroughly washed cells. Furthermore, the detection limit for [**1**](PF_6_)_2_ and [**3**](PF_6_)_2_ was determined to be below the applied concentration range (i.e., below 1 μm, data not shown). Unlike for ICP‐MS, partial speciation was possible with this technique. MALDI‐MS detects monocationic species,^12^ and in the particular case of [**1**]^2+^ leads to the detection of a unique signal at *m*/*z* 490.1,^13^ which can be assigned to [Ru(tpy)(bpy‐H)]^+^ (Figures S6 and S7 in the Supporting Information). Because MALDI‐MS utilizes UV laser ionization (*λ*_exc_=355 nm), the ionization of [**3**](PF_6_)_2_ was followed by photosubstitution of ligand **2** and detection of the same signal at *m*/*z* 490.1; in other words, it was impossible to distinguish [**3**]^2+^ from [**1**]^2+^. However, MALDI‐MS conditions are much milder than those used for ICP‐MS analysis, that is, cells were not destroyed before the measurement and molecules were not atomized during ionization. As a consequence, it was possible to compare the proportion of cell‐based signals to that of ruthenium‐based signals (e.g., *m*/*z* 490.1), which were characterized by their unique isotope patterns.

**Figure 3:**
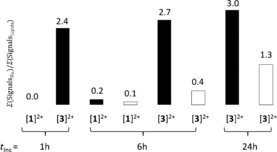
Ratio of Ru‐based/cell‐based signals observed by MALDI‐MS after different cell treatment conditions. Black bars correspond to samples kept in the dark, white bars to irradiated cells (*λ*=454 nm, 10 min, 6.3 J cm^−2^). Incubation times before refreshing of medium and irradiation are indicated.

The ratio of ruthenium to cell signal in MALDI‐MS was used to compare drug uptake (see Figure 3 and Table S2 in the Supporting Information for details). As shown in Figure 3, incubation with complex [**1**](PF_6_)_2_ resulted in negligible ruthenium signals, whereas incubation with [**3**](PF_6_)_2_ resulted in a significant, time‐dependent increase in the ratio of Ru/lipid signals. The observed difference between [**1**](PF_6_)_2_ and [**3**](PF_6_)_2_ is in good correlation with their different hydrophobicities,^14^ and indicates different drug–cell interactions. The less hydrophobic ruthenium–polypyridyl complex [**1**]^2+^ was seemingly washed away from the cells, which suggested that [**1**](PF_6_)_2_ did not enter the cells, and/or interacted minimally with them. This result correlates with the low toxicity of this compound. In contrast, the cholesterol‐containing compound [**3**](PF_6_)_2_ was already found in higher quantities in the cells after 1 h of incubation, which indicated increased drug uptake compared with [**1**](PF_6_)_2_.

For cells treated with [**3**](PF_6_)_2_, the influence of light irradiation, which is accompanied by the in vitro formation of [**1**]^2+^, is indicated by the white bars in Figure 3. The ruthenium‐based MALDI‐MS intensity significantly decreased after light irradiation (see also Figure S7 in the Supporting Information in comparison with Figure S6). If the ruthenium species remained inside the cell after irradiation, and thus, were not washed away, a similar value for the ruthenium signals would have been expected under dark and irradiated conditions. Surprisingly, 85 % of the ruthenium‐based signals disappeared when light irradiation was performed after 6 h of incubation, and roughly 50 % when it was performed after 24 h of incubation. These results implied that after 6 h of incubation the light‐induced ligand exchange reaction shown in Figure 1 a released [**1**]^2+^ outside the cell into the medium, which was washed away before the MALDI‐MS analysis was performed. Therefore, compound [**3**]^2+^ must stick initially in the biological membrane with the cholesterol ligand inserted in the outer leaflet of the cell membrane, and the ruthenium ion pointing into the media. The higher ruthenium intensity observed when light irradiation was performed after a longer (24 h) incubation time further implied that [**3**](PF_6_)_2_ flipped in a time‐dependent manner into the inner leaflet of the cell membrane, probably in a similar but slower manner to that for cholesterol.^15^ These observations are consistent with the negligible effect of light irradiation after 6 h. It is only when the ruthenium complex has flip‐flopped towards the cytosol (i.e., after 24 h) that light irradiation, and with it the intracellular formation of [**1**]^2+^ (the “bee”), leads to additional cytotoxicity by one of the (unknown) intracellular interactions with DNA or proteins.

**Figure 4:**
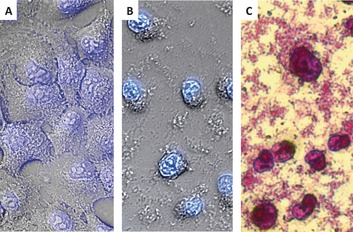
Micrographs of A549 cells (40×). 4′,6‐Diamidino‐2‐phenylindole (DAPI) staining (of DNA) of A) untreated cells and B) cells treated with [**3**](PF_6_)_2_ (25 μm). C) Micrograph of cells after treatment with [**3**](PF_6_)_2_ (25 μm), fixation (trichloroacetic acid (TCA), 10 %), and SRB staining (to visualize proteins).

The unspecific cytotoxicity in the dark, however, cannot be explained with this model. A more collective mode of action, similar to that of a swarm of bees, was found to be responsible for the cytotoxicity of [**3**](PF_6_)_2_ at higher concentrations in the dark. First, standard chemical biological experiments were conducted to analyze the type of cell death. A DNA ladder experiment (Figure S9 in the Supporting Information) showed that [**3**](PF_6_)_2_ acted independently of caspases.^16^ Second, cell cycle analysis (Figure S10 in the Supporting Information) confirmed that [**3**](PF_6_)_2_ did not interfere with proliferation,^9^, ^17^ and third, dye‐exclusion assays^18^ implied a concentration‐dependent loss of cell‐membrane integrity (Figure S11 in the Supporting Information). All these observations indicated that a necrotic form of cell death was induced by [**3**](PF_6_)_2_ at 10 μm in the dark. This hypothesis was confirmed by a flow cytometric annexin V‐propidium iodide assay (Figure S12 in the Supporting Information).^19^ In addition, optical microscopy imaging revealed an unusual cell‐size modulating activity of [**3**](PF_6_)_2_. With increasing concentrations, the cells shrunk to the size of the nuclei, without losing the outer cell membrane (Figure 4). In addition, significant debris was observed at concentrations of [**3**](PF_6_)_2_ higher than 10 μm. These debris particles could be stained with the protein‐binding dyes SRB^9^ (Figure 4 c) and trypan blue18b (Figure S11D in the Supporting Information), but not with the DNA‐specific dye DAPI.^20^, 4

**Figure 5:**
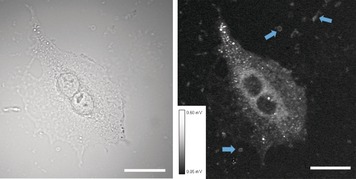
A) Bright‐field micrograph of A549 cells (32×) treated with [**3**](PF_6_)_2_ (10 μm). B) Stimulated Raman scattering (SRS) image of the same cell at $\tilde{\nu }$ =2850 cm^−1^, primarily selective for lipids. Blue arrows indicate some of the lipid‐containing debris particles. The intensity scale for the SRS signal (8‐bit) is given as inset. The scale bar is 20 μm.

Stimulated Raman scattering (SRS) microscopy experiments^21^ were conducted to look for the presence of lipids in those debris particles. Briefly, SRS signals were measured in living cells by utilizing the lipid‐specific CH_2_ stretching vibration at $\tilde{\nu }$ =2850 cm^−1^ (an example is depicted in Figure 5 B). The investigated cells showed the typical lipid distribution: a bright signal for the cell membrane, endoplasmic reticulum, Golgi apparatus and endosomal compartments, but no signal in the lipid‐poor nuclear region.^22^ The debris particles, which formed after treatment with high concentrations of [**3**](PF_6_)_2_, were already visible in the bright‐field image (Figure 5 A), and were between 1.0–2.5 μm in size. In the lipid‐sensitive SRS experiments, this debris gave a strong resonance signal (indicated with arrows in Figure 5 B). Altogether, these results implicated that the debris particles were lipid–protein aggregates extracted from the cell membrane, which explained why [**3**](PF_6_)_2_ induced a cell‐line‐unspecific, DNA‐independent cell death above 5 μm. Detergents, such as sodium dodecyl sulfate (SDS), Triton‐X 100, or cetyltrimethylammonium bromide (CTAB), are also known for their ability to extract lipid–protein aggregates from cell membranes and are visible as debris particles (Figure S8 in the Supporting Information).^23^ Thus, we hypothesized that [**3**](PF_6_)_2_, which consists of a charged ruthenium polypyridyl head group and a lipophilic tail, might behave as a metal–organic surfactant capable of aggregating above a certain concentration and affecting the cell membrane.5

To confirm this hypothesis, the critical aggregate concentration (CAC) of [**3**](PF_6_)_2_ was measured by dynamic light scattering (DLS).^23^ The CAC of a compound is the concentration at which further addition of amphiphilic molecules does not change the monomer concentration. Above this concentration, the monomer molecules are in equilibrium with supramolecular aggregates of finite size.^24^ A CAC of 3.5 (±0.5) μm was found for [**3**](PF_6_)_2_ (Figures S13 and S14 in the Supporting Information), which is a prototypical characteristic of molecular detergents.^25^ Thus, complex [**3**](PF_6_)_2_ is able to form aggregates of typically 68 (±10) nm (*z*_av_, according to DLS), which, in contact with cells, are susceptible to form mixed assemblies that also contain cellular lipids and proteins, in analogy to non‐metalated surfactants.^23^, ^26^

Additional experiments were conducted to gain a deeper understanding of these interactions. First, the time evolution of the A549 cell population treated with [**3**](PF_6_)_2_ was studied and compared with those for cisplatin, staurosporine, Triton‐X, and SDS. As shown in Figure S5 in the Supporting Information, after compound withdrawal, the cell population initially treated with [**3**](PF_6_)_2_ recovered, as observed for Triton‐X 100 or SDS. In contrast, for cisplatin‐ or staurosporine‐treated cells, no recovery was observed after withdrawing the drug‐loaded media. The recovery of cell proliferation for cells treated with [**3**](PF_6_)_2_ correlated well with cell cycle analysis by means of flow cytometry (Figure S10 in the Supporting Information), in which no difference between the control and the treated cell populations was found 24 h after media refreshment.

In a separate experiment, the dependence of the cytotoxicity (EC_50_) on the cell population was measured. As shown in Figure S15 in the Supporting Information, the EC_50_ of [**3**](PF_6_)_2_ decreased with an increased number of cells, whereas changing the cell density did not influence the EC_50_ of cisplatin. Thus, an increased number of cell membranes diminishes the toxicity of [**3**](PF_6_)_2_. Taken together, these facts are consistent with the hypothesis of a thermodynamically driven mode of action for high concentrations of [**3**](PF_6_)_2_ in the dark.

## Conclusion

**Figure 6:**
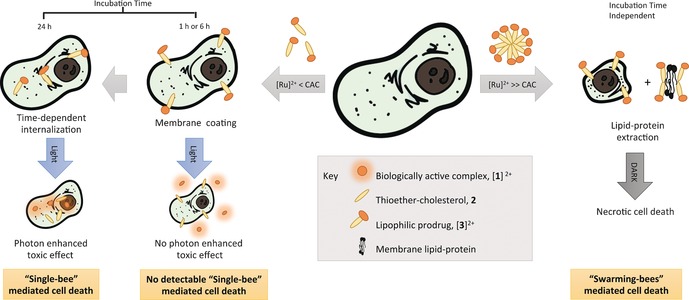
Proposed mode of action of [**3**](PF_6_)_2_. Treatment with concentrations above the CAC leads to lipid–protein extraction, and eventually to necrotic cell death. Treating the cells with concentrations below the CAC leads to insertion of the compound in the cell membrane, followed by a time‐dependent internalization. A significant photon‐enhanced effect was only observed after 24 h incubation, that is, after internalization of [**3**]^2+^, and thus, when the resulting active complex [**1**]^2+^ could interact with an (unknown) intracellular target.

Our understanding of the cytotoxic activity of [**3**](PF_6_)_2_ relies on two separate modes of action (Figure 6). At low concentrations relative to the CAC, monomers of [**3**](PF_6_)_2_, like single bees, insert quickly into the outer leaflet of the cell membrane. The flip‐flop to the inner leaflet, which leads to internalization, occurs in a slower manner. After a short (6 h) incubation time, the light‐induced release of membrane‐impermeable species [**1**]^2+^ occurs outside the cell without biological consequences. After a prolonged incubation time (24 h), the same photoreaction leads to the release of [**1**]^2+^ inside the cell, where an unknown target is reached that may coordinate to ruthenium, eventually resulting in a more lethal signal. This first mode of action is similar to that of recently reported ruthenium compounds.^27^ At concentrations above the CAC of (3.5±0.5) μm, however, complex [**3**](PF_6_)_2_ most likely behaves like a swarm by forming aggregates. When the ratio between these aggregates and the cell membrane lipids is high enough, thermodynamic forces lead to the generation of holes in the cell membrane (Figure S11 in the Supporting Information) and, at the highest concentrations tested (10–25 μm), to lipid–protein extraction of the cell membrane and formation of ternary aggregates containing cell lipids, membrane proteins, and [**3**](PF_6_)_2_ (Figures 4 and 5 and Figure S8 in the Supporting Information). Because it is simply based on the lipid/detergent equilibrium, this second mode of action is neither cell‐line specific, nor enhanced by light irradiation. On the contrary, light irradiation transforms [**3**]^2+^ back to ligand **2** and the aqua complex [**1**]^2+^, that is, it destroys the amphiphilic character of [**3**]^2+^ and its ability to form toxic aggregates.

Such a bifaceted mode of action is unprecedented among metallodrugs, which are usually supposed to target nuclear DNA, mitochondrial membranes, or proteins. The complex behavior of compound [**3**](PF_6_)_2_ is due to the combination of its amphiphilic character and light sensitivity. This type of compound opens up two separate roads for drug delivery. First, as a monomer they can be used as prodrugs to release the toxic part (herein [**1**]^2+^) by light irradiation. Second, as aggregate‐forming molecules they may be used as light‐sensitive drug carriers that, similar to a Trojan horse, can release a lipophilic load under visible‐light irradiation, which annihilates its amphiphilic character. In addition, these results should serve as a warning for the design of future metallodrugs. Increasing lipophilicity is often used as a way to increase cellular uptake.^28^ However, we showed herein that, by combining a charged metal‐based head with a fatty tail, self‐aggregation became possible, which radically changed not only how much of the compound penetrated into the cell, but also the mode of action of the compound. Finally, the possibility of extracting lipid–protein aggregates with an inorganic surfactant may offer significant advantages in the field of proteomics and lipidomics, due to the unique methods (e.g., MALDI‐MS) available for detecting inorganic compounds.

## Experimental Section

### General

^1^H NMR spectra were recorded by using a Bruker DPX‐300 spectrometer; chemical shifts are indicated in ppm relative to tetramethylsilane (TMS). Electrospray mass spectra were recorded on a Finnigan TSQ‐quantum instrument by using an ESI technique. DLS was performed with a Nanosizer instrument from Malvern operating at *λ*_irr_=633 nm. A Tecan M1000 PRO plate reader was used for fluorescence and absorbance measurements in 96‐well plates. Images and data were processed with Origin Pro, FCS Express, Prism 5.0, ChemDraw, Gimp 2.0, and Microsoft Excel software.

### Synthesis and crystal growth

The synthesis of all described ligands and complexes was performed as reported previously. 3β‐(2‐{2‐[2‐(Methylthio)ethoxy]ethoxy}ethoxy)cholesterol (**2**; CAS‐Nr: 1373125‐91‐7) was described by Bahreman et al.5d [Ru(tpy)(bpy)(Cl)]Cl and [**1**](PF_6_)_2_ were synthesized according to previous reports,5b, 29 and [**3**](PF_6_)_2_ was prepared as described by Askes et al.7a Single crystals of [**3**](PF_6_)_2_ were obtained by slow recrystallization through the vapor diffusion of diethyl ether into a solution of the complex in ethyl acetate. Long and thin crystals with a ruby color were obtained that were suitable for X‐ray crystal structure determination.

### Single‐crystal X‐ray crystallography

All reflection intensities were measured at 110(2) K by using a SuperNova diffractometer (equipped with an Atlas detector) with Cu_Kα_ radiation (*λ*=1.54178 Å) under the program CrysAlisPro (Version 1.171.36.32 Agilent Technologies, 2013). The program CrysAlisPro (Version 1.171.36.32 Agilent Technologies, 2013) was used to refine the cell dimensions and for data reduction. The structure was solved with the program SHELXS‐2013^30^ and was refined on *F* ^2^ with SHELXL‐2013 (Sheldrick, 2015). Analytical numeric absorption corrections based on a multifaceted crystal model were applied by using CrysAlisPro (Version 1.171.36.32 Agilent Technologies, 2013). The temperature of the data collection was controlled by using the system Cryojet (manufactured by Oxford Instruments). The H atoms were placed at calculated positions by using the instructions AFIX 13, AFIX 23, AFIX 43, or AFIX 137 with isotropic displacement parameters with values 1.2 or 1.5 times *U*eq of the attached C atoms.

The structure was partly disordered. The fragment C52B→C59B/C52′→C59’ (C53B excluded) was disordered over two orientations, and the occupancy factor of the major component of the disorder was refined to 0.561(7). The contribution of two disordered ethyl acetate solvent molecules was removed from the final refinement by using SQUEEZE5f (details are provided in the CIF file). The absolute configuration was established by anomalous dispersion effects in diffraction measurements on the crystal. The Flack parameter refined to 0.009(7).

**Crystal data for [3](PF_6_)_2_**: *M*_r_=1329.34; orange–red plates; 0.48×0.38×0.02 mm^3^; monoclinic; *P*2_1_ (no. 4); *a*=37.5901(3), *b*=10.59806(7), *c*=16.68304(13) Å; *β*=100.6353(8)°; *V*=6532.05(9) Å^3^; *Z*=4; *D*_x_=1.352 g cm^−3^; *μ*=3.389 mm^−1^; *T*_min_−*T*_max_: 0.341–0.935; 77 205 reflections were measured up to a resolution of (sin *θ*/*λ*)_max_=0.62 Å^−1^; 22 930 reflections were unique (*R*_int_=0.0464), of which 21 312 were observed [*I>*2σ(*I*)]; 1552 parameters were refined by using 223 restraints; *R*1/*wR*2 [*I>*2σ(*I*)]: 0.0387/0.1017; *R*1/*wR*2 (all reflns): 0.0422/0.1046; *S*=1.035; residual electron density was found between −0.47 and 0.64 e Å^−3^.

CCDC‐1430105 contains the supplementary crystallographic data for this paper. These data are provided free of charge by The Cambridge Crystallographic Data Centre.

### Stability assays

**In a mixture of [D_6_]DMSO/PBS (7:1)**: Complex [**3**](PF_6_)_2_ was dissolved in a 7:1 mixture of [D_6_]DMSO/phosphate‐buffered saline (PBS) mixture and immediately subjected to ^1^H NMR spectroscopy measurements (300 MHz, 128 scans). The tube was placed in an incubator set at 37 °C, and additional spectra were measured after 26, 49, and 73 h in the dark. No changes could be measured (see Figure S1 in the Supporting Information), which proved that [**3**](PF_6_)_2_ was thermally stable under such conditions.

**In Opti‐MEM**: The thermal stability of complexes [Ru(tpy)(bpy)Cl]Cl, [**1**](PF_6_)_2_, and [**3**](PF_6_)_2_ under cell culture conditions (Opti‐MEM complete, 37 °C) was investigated by measuring the evolution of the UV/Vis spectra in the dark by using a Tecan M1000PRO reader (Figure S2 in the Supporting Information). After 6 h, the first two compounds had reacted with medium components, whereas [**3**](PF_6_)_2_ was essentially unchanged.

### Cell culturing: Reagents and cells

Cells (A‐375, human malignant melanoma; A‐431, human epidermoid carcinoma; A549, human lung carcinoma; MCF7, human mammary gland adenocarcinoma; MDA‐MB‐231, human mammary gland adenocarcinoma; U‐87 MG, human glioblastoma grade IV) were distributed by the European Collection of Cell Cultures (ECACC), and purchased through Sigma Aldrich. Dulbecco's modified Eagle medium (DMEM, with and without phenol red, without glutamine), 200 mm glutamine‐S (GM), TCA, glacial acetic acid, SRB, and tris(hydroxylmethyl)aminomethane (tris base) were purchased from Sigma Aldrich. Fetal calf serum (FCS) was purchased from Hyclone. Penicillin and streptomycin were purchased from Duchefa and were diluted to a 100 mg mL^−1^ solution of penicillin/streptomycin (P/S). Trypsin and Opti‐MEM^®^ (without phenol red) were purchased from Gibco^®^ Life Technologies. Trypan blue (0.4 % in 0.81 % sodium chloride and 0.06 % potassium phosphate dibasic solution) was purchased from BioRad. Plastic disposable flasks and 96‐well transparent plates were obtained from Sarstedt. The 96‐well black plates were from Greiner Bio‐one (5665–5090). Eight‐chamber microscope slides, Nunc^®^ Lab‐Tek^®^ II Chamber slide^TM^ systems, were purchased from Sigma Aldrich. RNAse A and proteinase K (from Tritirachium album) were purchased from Sigma Aldrich. The 1‐kb Plus DNA ladder was purchased from Fisher Scientific. Tris‐acetate–ethylenediaminetetraacetic acid (EDTA; TAE) buffer (pH 8) was prepared as a 50× stock solution and diluted to 1× prior to usage. DNA‐loading buffer was prepared as a 10 mL stock solution by using glycerol (3 mL), a solution of EDTA (1 mL, 0.5 m, pH 8), and methylene blue (5 mg). Cell lysis buffer (100 mm Tris‐HCl, pH 8, 20 mm EDTA, 0.8 % SDS) was freshly prepared. Mass spectrometry experiments were performed on a Synapt G2‐Si MALDI‐TOF mass spectrometer (Waters Corporation, Milford, MA), equipped with a *λ*=355 nm laser. Before the measurements, the instrument was calibrated by using red phosphorus (Acros Organics). A 0.5 m solution of 2,5‐dihydroxybenzoic acid (DHB; Sigma) in methanol was used as a matrix. Mass spectra were acquired in positive mode.

### General cell culturing

Cells were purchased and upon receipt were cultured for working and frozen stocks. Each cell line was cultured in DMEM complete, with phenol red, supplemented with 8.0 % v/v FCS, 0.2 % v/v P/S, and 0.9 % v/v GM. Cells were cultured in 25 cm^2^ flasks and were split into a new passage at 70–80 % confluence (approximately 3× per week). Flasks were incubated at 37 °C with a CO_2_ level of 7.0 %. Media was changed every second day. For all irradiation experiments, Opti‐MEM^®^ media, supplemented with 2.5 % FCS, 0.2 % v/v P/S, and 1 % v/v GM (later on called OMEM complete) was used. Cells were passaged for 4–8 weeks. SRB was purchased from Alfa Aesar GmbH&Co under the tradename Kiton Red S.

### 1 ‐LED irradiation setup

A 96 light‐emitting diode (LED) array, described in depth by Hopkins et al.,^8^ allowed the irradiation of a 96‐well plate at 37 °C and, in parallel, maintained dark control under otherwise identical conditions. The light of the LED array had a wavelength of *λ*_max_=(454±22) nm, and at a voltage of 28.9 V the power at the bottom of each well was (10.5±0.7) mW cm^−2^.

### Cell‐free light irradiation of [3](PF_6_)_2_ in 96‐well plates

In a 96‐microtiter black plate, solutions of [**3**](PF_6_)_2_ (25 μm) in Opti‐MEM complete media (200 μL) were irradiated in triplicate at 37 °C by using the LED setup. After 20, 15, 12, 10, 8, 6, 4, 2, and 0 min of irradiation, the UV/Vis spectrum of each well was measured by using a Tecan M1000pro plate reader (Figure S3 in the Supporting Information). The samples were submitted to ESI‐MS measurements to confirm the light‐induced release of ligand **2** from the complex upon irradiation in Opti‐MEM complete (Figure S4 in the Supporting Information). Overall, under cell‐growing conditions, 8 min or 5 J cm^−2^ of blue‐light irradiation was enough to almost fully activate 5 nmol of the Ru complex [**3**]^2+^.

### Dark cytotoxicity and phototoxicity on human cancer cell lines

The cytotoxicity of compounds **2**, [Ru(tpy)(bpy)(Cl)]Cl, [**1**](PF_6_)_2_, and [**3**](PF_6_)_2_ was evaluated by using the SRB microculture colorimetric assay. In short, exponentially growing cells were seeded in Opti‐MEM^®^ (without phenol red, w/2.5 % FCS, P/S, and GM) into 96‐well plates at *t*=0 at the appropriate cell densities (A375=7000 cells/well, A431=8000 cells/well, A549=5000 cells/well, MCF‐7=8000 cells/well, MDA‐MB‐231=12 000 cells/well, U87Mg=6000 cells/well) to prevent confluence of the cells during the experiment. At *t*=6 or 24 h, the cells were treated with serial dilutions of each compound in Opti‐MEM, depending on the expected EC_50_ value after 6 or 24 h of incubation. For **2**, [Ru(tpy)(bpy)(Cl)]Cl, and [**1**](PF_6_)_2_, the concentration series was 1.50, 7.50, 15.00, 30.00, 75.00, and 150.00 μm; for [**3**](PF_6_)_2_ it was 0.25, 1.25, 2.50, 5.00, 12.50, and 25.00 μm. The final DMSO concentration per well never exceeded 0.75 %, which was nontoxic to the cells.^31^ After 6 or 24 h of incubation with the drug‐loaded media, the media was aspirated and replaced by fresh, warm (37 °C) media; the plates were kept on a heating mat during media refreshing to avoid a significant drop in temperature for the cells, and to insure a temperature of at least 33 °C during light irradiation. Light irradiation was performed for 10 min (*λ*=454 nm, 6.3 J cm ^−2^) by using the LED‐based, 96‐well plate irradiation setup described by Hopkins et al.^8^ A duplicate plate was treated the same way, but without light irradiation and was further referred to as the dark control.

The percentages of surviving cells relative to compound‐free wells were determined 72 h after the beginning of drug exposure, that is, at *t*=96 h, by using the SRB assay.^9^ Briefly, cells were fixed by using cold TCA (10 % w/v) and maintained at 4 °C for 4–48 h. Once fixed, TCA was removed from the wells, plates were gently washed 5× with water, air dried, stained by using 100 μL SRB (0.6 % w/v SRB in 1 % v/v acetic acid) for 30–45 min, washed with approximately 300 μL acetic acid (1 % v/v) 5× times, air dried, and the dye was then solubilized by using 10 mm tris base. The absorbance at *λ*=510 nm was read by using a M1000 Tecan Reader. The SRB absorbance data were used to evaluate the viable cell population in Excel and GraphPad Prism. The absorbance data from three wells (technical replicates, *n*_t_=3) for each cell line and concentration were averaged. Relative cell populations were calculated by dividing the average absorbance of the irradiated wells by the average absorbance of the dark control. Three biological replicates (*n*_b_=3) of each treatment and cell line were completed. The averages of the biological replications were plotted as relative cell population versus log (concentration in μm) with standard error of each concentration. For each cell line, the EC_50_ was calculated by fitting the logarithmic dose–response curves through nonlinear regression with a fixed *Y* maximum (100 %) and minimum (0 %) relative cell population, and a variable Hill slope; this resulted in the simplified two parameter Hill slope equation by using PRISM 5.0.

### MALDI‐MS

Qualitative uptake experiments were performed through MALDI‐MS experiments. Cells of cell‐line A549 were seeded in Opti‐MEM^®^ (without phenol red, w/2.5 % FCS, P/S, and GM) in an eight‐chamber glass slide (25 000 cells/well). Treatment with solutions of [**1**](PF_6_)_2_, [**3**](PF_6_)_2_, or [Ru(tpy)(bpy)(Cl)]Cl for 24 h, 6 h, 1 h, or 1 min was performed 24–48 h after seeding. After incubation with the drug, the supernatant media was aspirated, the cells were washed gently 3× with PBS, fresh media was added, and the cells were irradiated with blue light by using the same 96‐LED array as that used for irradiating 96‐well plates (*λ*=455 nm, 10 min, 6.3 J cm^−2^, 37 °C). An identical eight‐chamber slide was prepared, but left in the dark as a control. After removing the chamber (on top of the glass slide) and drying the cell monolayer under ambient conditions, a 0.5 m solution of DHB matrix in methanol was applied by means of a pipette, and the samples were submitted to the MALDI SYNAPT G2‐Si mass spectrometer.

To analyze the data, the individual signal height of the cell and the drug‐specific signals were first measured. Because the mass spectra from the untreated cell culture (control) showed a different pattern, depending on the treatment (light irradiated vs. dark probe, compare Figure S6 A vs Figure S7 A* in the Supporting Information), several lipid signals were chosen to decrease a potential bias due to irradiation. After summing up the height of the cell‐ and drug‐specific signals, the ratio between the heights was calculated to analyze the distribution of the investigated sample, and to eventually be able to determine the uptake indirectly. In Table S2 in the Supporting Information, the list of chosen signals and assignments, as far as possible, is given.

### Microscopic investigation of living cells in the presence of surfactants in the dark

Cells were seeded in a 96‐well plate according to the cytotoxicity assay. After 24 h of incubation, the medium was removed and the cells were treated with increasing concentrations of the indicated drugs in Opti‐MEM complete. Optical microscopy images were recorded after 24 h of drug incubation in the dark (37 °C, 7 % CO_2_) at the indicated magnifications.

**Dye‐exclusion assay**: The supernatant media was removed, the cells were treated with a diluted solution of trypan blue (0.25 %), and the cells submitted to microscopic investigations (see Figure S11 in the Supporting Information).

**DAPI staining**: The supernatant media was removed, the cells were stained for 10 min with a solution of DAPI (0.01 mg mL^−1^), and then the cells were imaged.

### DNA‐laddering experiment

Approximately 500 000 cells of the cell‐line A549 were seeded in cell culture flasks (25 cm^2^) and grown in DMEM (10 % FCS, 0.2 % P/S, 0.9 % GM). After 24 h, the supernatant media was removed and the nonconfluent cell monolayer was reloaded with substance‐loaded medium (or a blank fresh medium as a control). After 24–72 h, the supernatant medium was collected and the cell monolayer was washed with PBS. The combined media and PBS were centrifuged (1500 rpm, 5 min, Eppendorf 5702 centrifuge). The pellet of dead cells was gently suspended in PBS (1 mL) and centrifuged again (1500 rpm, 5 min, 278 K, Eppendorf Centrifuge 5418). PBS was removed and lysis buffer (30 μL, 0 °C, 10 min) was added. Then RNAse (100 μg mL^−1^, 10 μL) was added and the cells were incubated for 10 min on ice followed by a prolonged incubation at 37 °C for 2 h. To finally digest the cell proteins, the cell pellet was treated with protein kinase K (10 μL) at 52 °C for 12 h. The extract was mixed with DNA‐ladder dye (10 μL) and analyzed by gel electrophoresis (2 % agarose loaded with 10 μL ethidium bromide (10 mg mL^−1^), 150 mV, 2 h, TAE buffer). The DNA bands were analyzed (see Figure S9 in the Supporting Information) by using a UV transilluminator (BioRad).

### Cell cycle investigation by flow cytometry

Approximately 500 000 cells of the cell‐line A549 were seeded in cell culture flasks (25 cm^2^) and grown in DMEM complete. After 24 h, the supernatant media was removed and the nonconfluent cell monolayer reloaded with substance‐loaded medium (or a blank fresh medium as a control). After 24–72 h of drug incubation, the supernatant medium was submitted to the DNA‐laddering experiment (see above), whereas the living cells were washed and harvested with trypsin. After centrifugation (1500 rpm, 5 min, Eppendorf Centrifuge 5702), the supernatant was withdrawn, the cells resuspended with PBS (1 mL), and centrifuged (1500 rpm, 5 min, Eppendorf Centrifuge 5418). After additional washing with PBS, the cells were fixed by adding ice‐cold ethanol (70 %) dropwise to the cells stored on ice. For thorough fixation and permeabilization, the cells were stored at least for 24 h at −20 °C. Thereafter, the cells were washed with PBS buffer (with Mg^2+^ and Ca^2+^, containing 1 % bovine serum albumin (BSA), and 0.1 % NaN_3_, 3×1 mL, 1000 rpm, Eppendorf Centrifuge 5418). Several cell suspensions were adjusted to the same cell concentration (ca. 100 000 cells mL^−1^), gently suspended in staining buffer (PBS buffer containing BSA, RNAse, NaN_3_, and PI analogue reported by Darzynkiewicz et al.^17^), and incubated for 30 min at room temperature in the dark. Analyses were performed by using a Beckman Coulter Quanta machine; collecting data from the FL2 channel. The cell population of interest was selected by plotting EV versus FL1. For each cell cycle distribution 100 000 events were collected. The distribution was calculated by using FCSexpress software by applying the method of Dean and Jett.^32^ Representative histograms and compiled results are shown in Figure S10 in the Supporting Information.

### SRS microscopy

The cell lipids were visualized by utilizing SRS microscopy. Cells of the cell‐line A549 were seeded in Opti‐MEM^®^ (without phenol red, w/2.5 % FCS, P/S, and GM) in an eight‐chamber glass slide (5000 cells/well). After 24 h, cells were treated with solutions of [**3**](PF_6_)_2_ (10 μm) for 24 h. After incubation with the drug, the supernatant media was aspirated, the cells were washed gently with PBS, and fresh media was added. Before SRS measurements, the media was aspirated, the chamber (on top of the glass slide) was removed, and a cover glass was mounted. The experimental setup for SRS imaging was mainly as described previously.21d In brief, laser light at *λ*=1064.4 nm (80 MHz, 8 ps) was intensity‐modulated at 3.636 MHz with an acousto‐optic modulator and overlapped with *λ*=816.7 nm light for imaging at $\tilde{\nu }$ =2850 cm^−1^. A Zeiss laser scanning microscope with a 32× objective (C‐Achroplan W, NA=0.85) was used to image samples with non‐descanned detection in forward scattering mode at 512×512 pixels with a pixel dwell time of 177 μs. Time‐averaged laser powers on the sample were 10 mW for the pump beam and 20 mW for the Stokes beam. The signal was amplified with a homebuilt transimpedance amplifier before demodulation in a lock‐in amplifier (SR844 Stanford Research Systems). The X‐phase output at a sensitivity of 1 mV was supplied to the ZEN microscopy software for image recording and processing.

### Determination of the CAC

The CAC was measured by using a fixed‐angle light‐scattering technique.25a, 33 Duplicates of a concentration series of [**3**](PF_6_)_2_ (0 to 10 μm) in distilled water were prepared and submitted to DLS measurements (Malvern Nanosizer, *λ*=633 nm). The attenuation factor was fixed to 11. For the analysis, the intensity of the scattered light (in kilocounts per second) was plotted against the concentration of [**3**](PF_6_)_2_ (see Figure S13 in the Supporting Information), according to an application note from Malvern.25b

## Supporting information

As a service to our authors and readers, this journal provides supporting information supplied by the authors. Such materials are peer reviewed and may be re‐organized for online delivery, but are not copy‐edited or typeset. Technical support issues arising from supporting information (other than missing files) should be addressed to the authors.

Supplementary
