# Supplementary material for: Chemical Swarming: Depending on Concentration, an Amphiphilic Ruthenium Polypyridyl Complex Induces Cell Death via Two Different Mechanisms
Source: Chemistry. 2016 Jul 4;22(31):10960–8. doi: 10.1002/chem.201600927 (PMC5096026; doi:10.1002/chem.201600927)
Supplement: Supplementary file 1 — Supplementary [file CHEM-22-10960-s001.pdf]

CHEMISTRY
A European Journal

Supporting Information

Chemical Swarming: Depending on Concentration, an Amphiphilic Ruthenium Polypyridyl Complex Induces Cell Death via Two Different Mechanisms

Bianka Siewert,[a] Vincent H. S. van Rixel,[a] Eva J. van Rooden,[a] Samantha L. Hopkins,[a] Miriam J. B. Moester,[b] Freek Ariese,[b] Maxime A. Siegler,[c] and Sylvestre Bonnet*[a]

chem_201600927_sm_miscellaneous_information.pdf

Electronic Supplementary Information

1. Table of content

1. Table of content 1
2. Stability in the dark in DMSO-d_6:PBS mixture (7:1) 2
3. Stability in the dark in Opti-MEM 2
4. Irradiation under *in vitro* conditions 3
5. ESI mass spectra in medium in the dark and under light irradiation 4
6. Photo Cytotoxicity 7
7. Growing curves of A549 cancer cell line in presence of different compounds 7
8. MALDI-MS analysis with cells 8
9. Microscopic investigation of living cells in the presence of surfactants in the dark 11
10. DNA-Laddering Experiment 11
11. Cell Cycle Investigation by Flow Cytometry 12
12. Dye-exclusion assay (trypan blue) 13
13. Annexin V-Propidium Iodid based Apoptosis assay. 13
14. Determination of the critical aggregate concentration (CAC) 14
15. Dose-response-curves of [3]$(PF_6)_2$ and CDDP treated cells and dependence on seeded cell numbers 15
16. References 15

2. Stability in the dark in DMSO-d_6:PBS mixture (7:1)

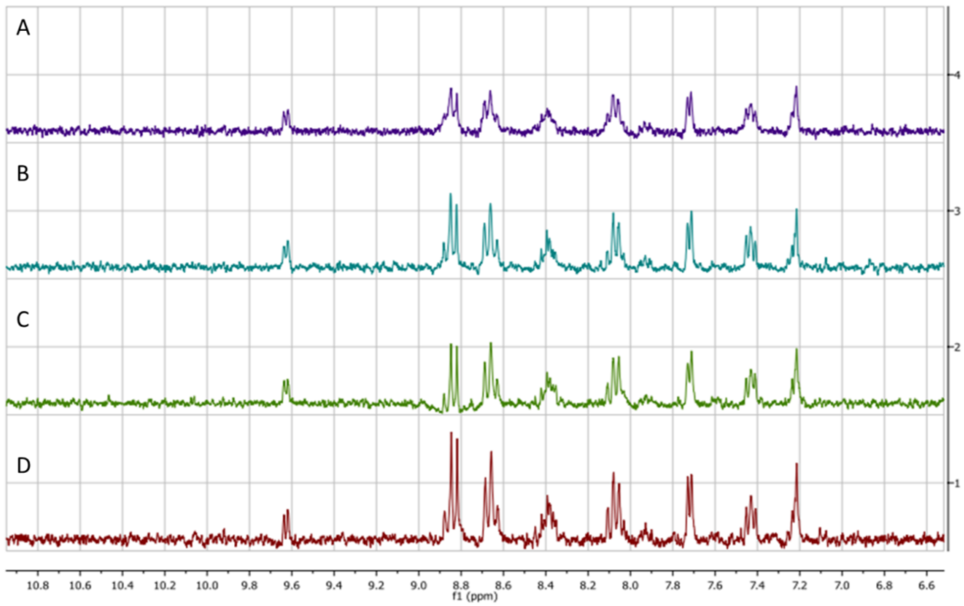

Figure S1.: ^{1}H-NMR spectra (300 MHz, aromatic region) of compound [**3**]$(PF_6)_2$ (125 µM) in DMSO:PBS (7:1) directly after solubilization (**A**), after 26 h (**B**), 49 h (**C**), and 73 h (**D**) in the dark at 37 ^{o}C.

3. Stability in the dark in Opti-MEM

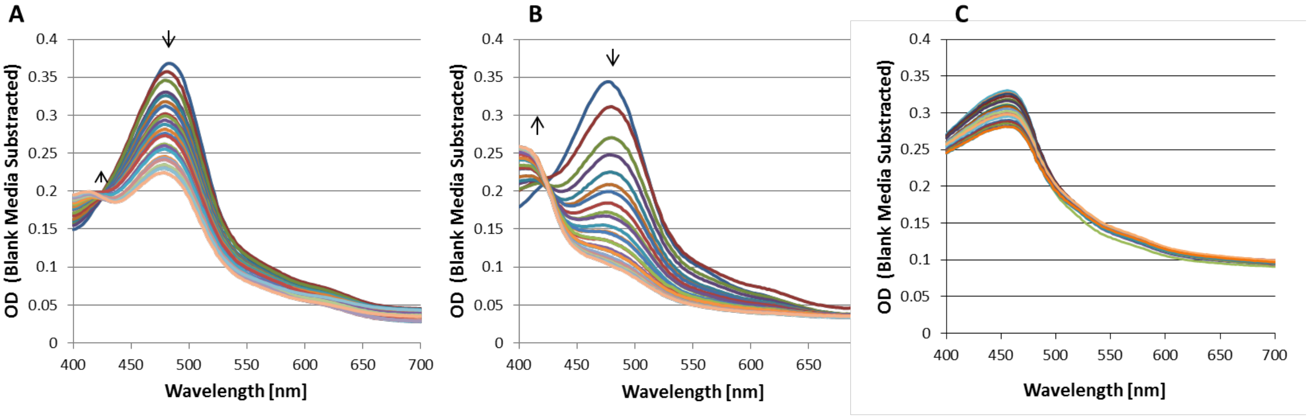

Figure S2. Evolution of the UV-vis spectra of [Ru(tpy)(bpy)Cl]Cl (**A**), [**1**]$(PF_6)_2$ (**B**), and [**3**]$(PF_6)_2$ (**C**) in Opti-MEM complete in the dark at 37 °C over a time period of 24 h. Baseline evolution is due to evaporation.

4. Irradiation under *in vitro* conditions

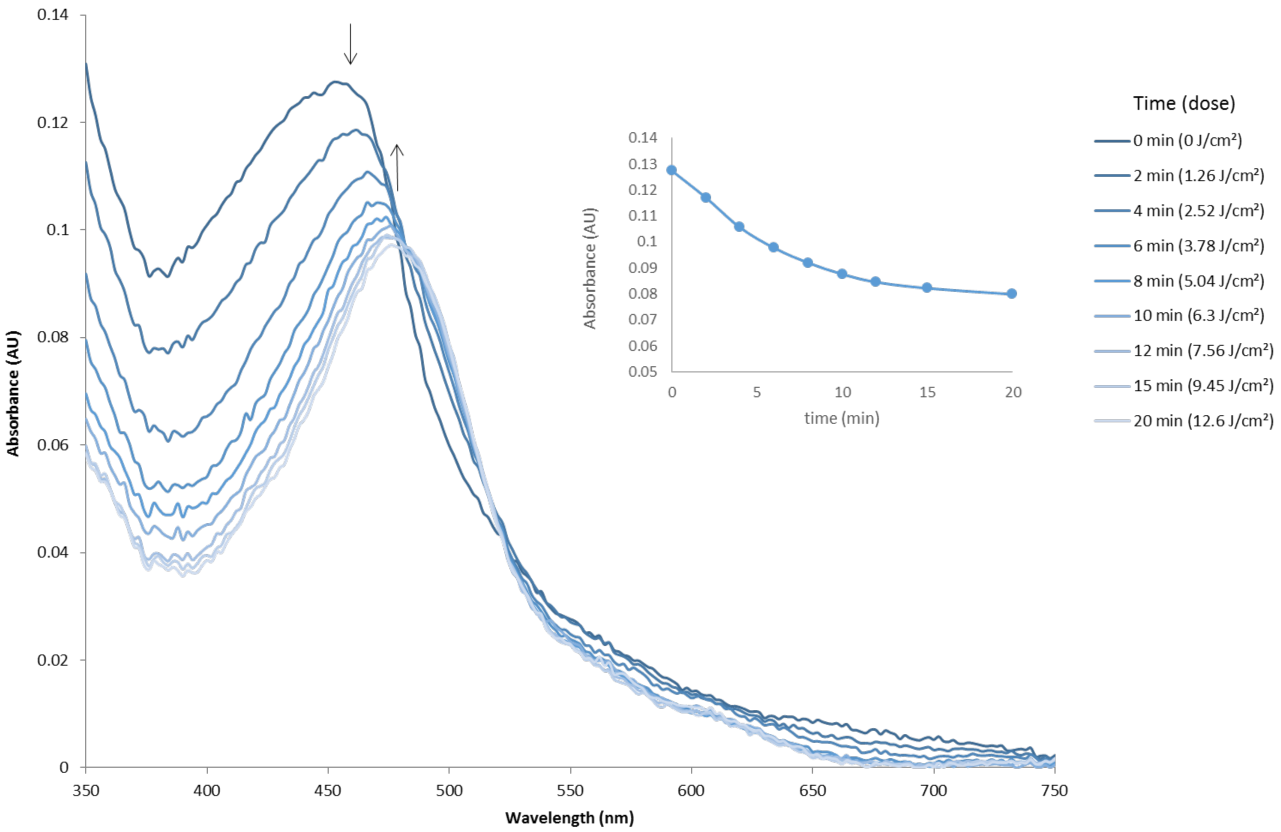

Figure S3. Irradiation of [**3**]$(PF_6)_2$ (25 µM) under cell-free *in vitro* conditions (96-well plate, 200 µL, in Opti-MEM complete, 37 ºC) using the blue LED irradiation setup (455 nm, 10.5 mW.cm^{-2}). After irradiating with different light doses (see legend) UV-vis spectra were measured with the Tecan M1000pro Plate reader. Irradiations were done in triplicate and the spectra shown represent the averages. The spectrum of the medium was subtracted from all other spectra. The spectral changes were practically complete after *ca*. 8 min (5 J.cm^{-2}) of irradiation at T = 310 K. The insert shows the decrease in absorbance over time at 454 nm.

5. ESI mass spectra in medium in the dark and under light irradiation

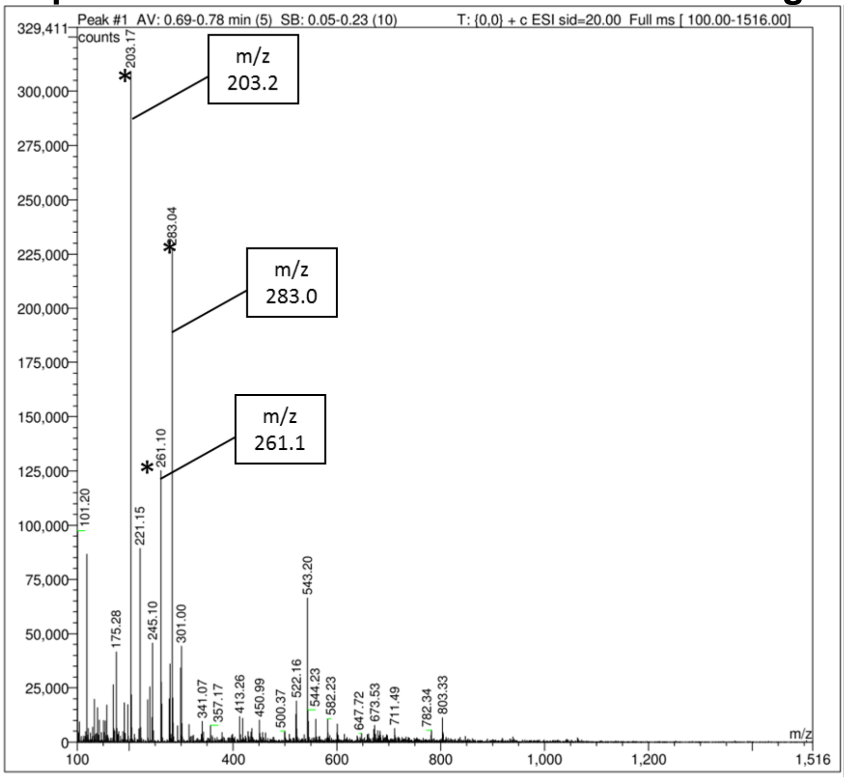

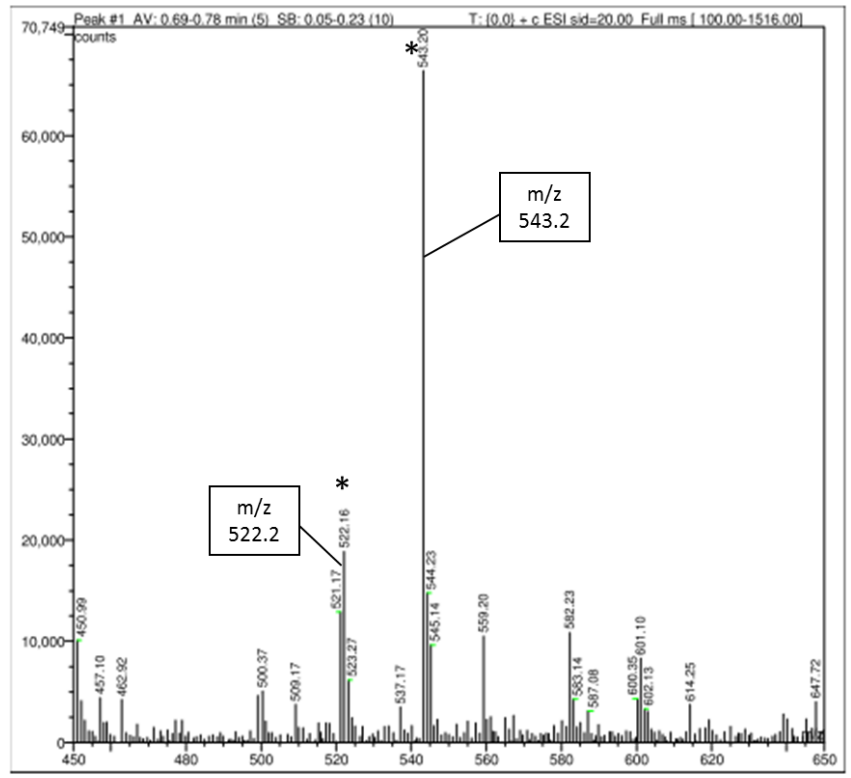

Figure S4A. ESI-MS spectra of Opti-MEM medium (2.5% FCS, Pen/Strep, GlutaMax) in the dark: full range (top) and zoom (bottom). Asterisks indicate the major medium-characteristic signals, which are labelled with their m/z value for clarity.

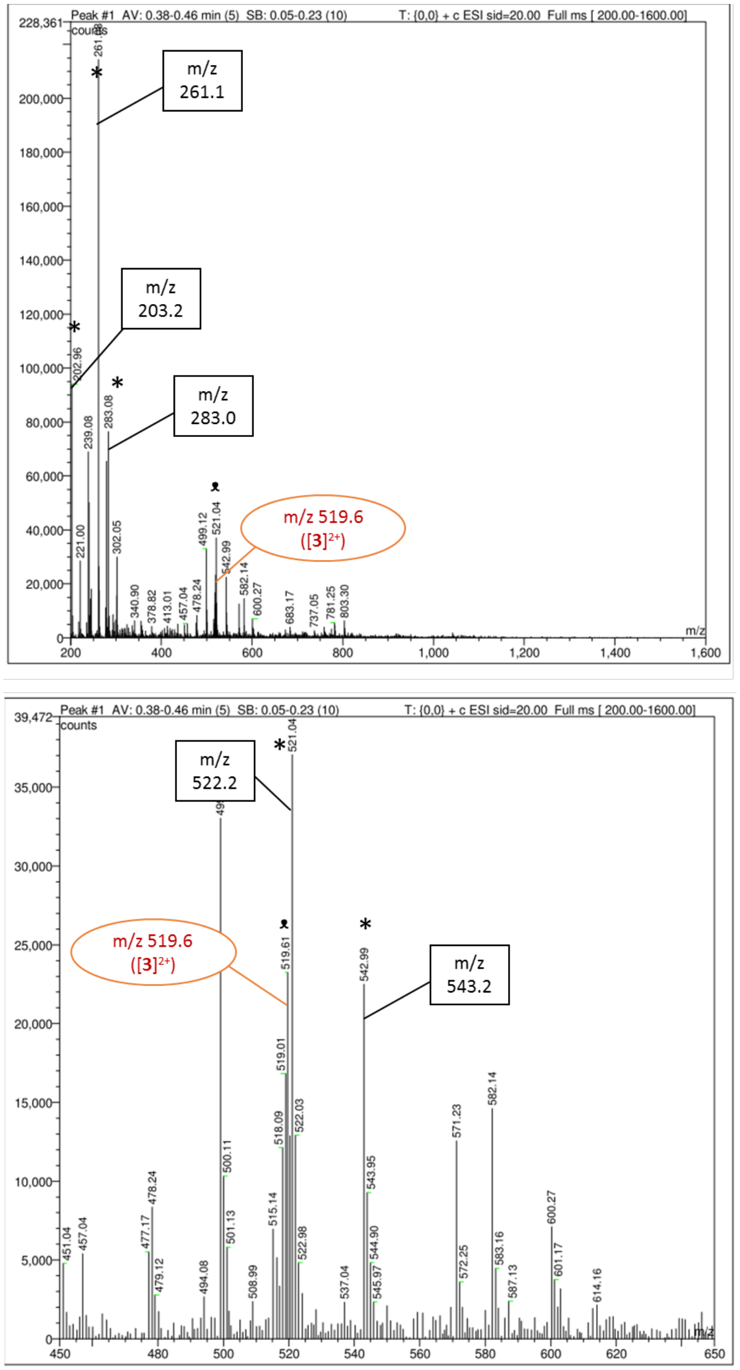

Figure S4B. ESI-MS spectra of [**3**]$(PF_6)_2$ (25 µM) in Opti-MEM medium (2.5% FCS, Pen/Strep, GlutaMax) in the dark: full range (top) and zoom (bottom). Asterisks indicate the medium-characteristic signals. * The peak at m/z = 519.6 stands for $[\mathbf{3}]^{2+}$ (doubly charged molecular ion, calc. m/z = 519.4). All relevant signals are labelled with their m/z values for clarity.

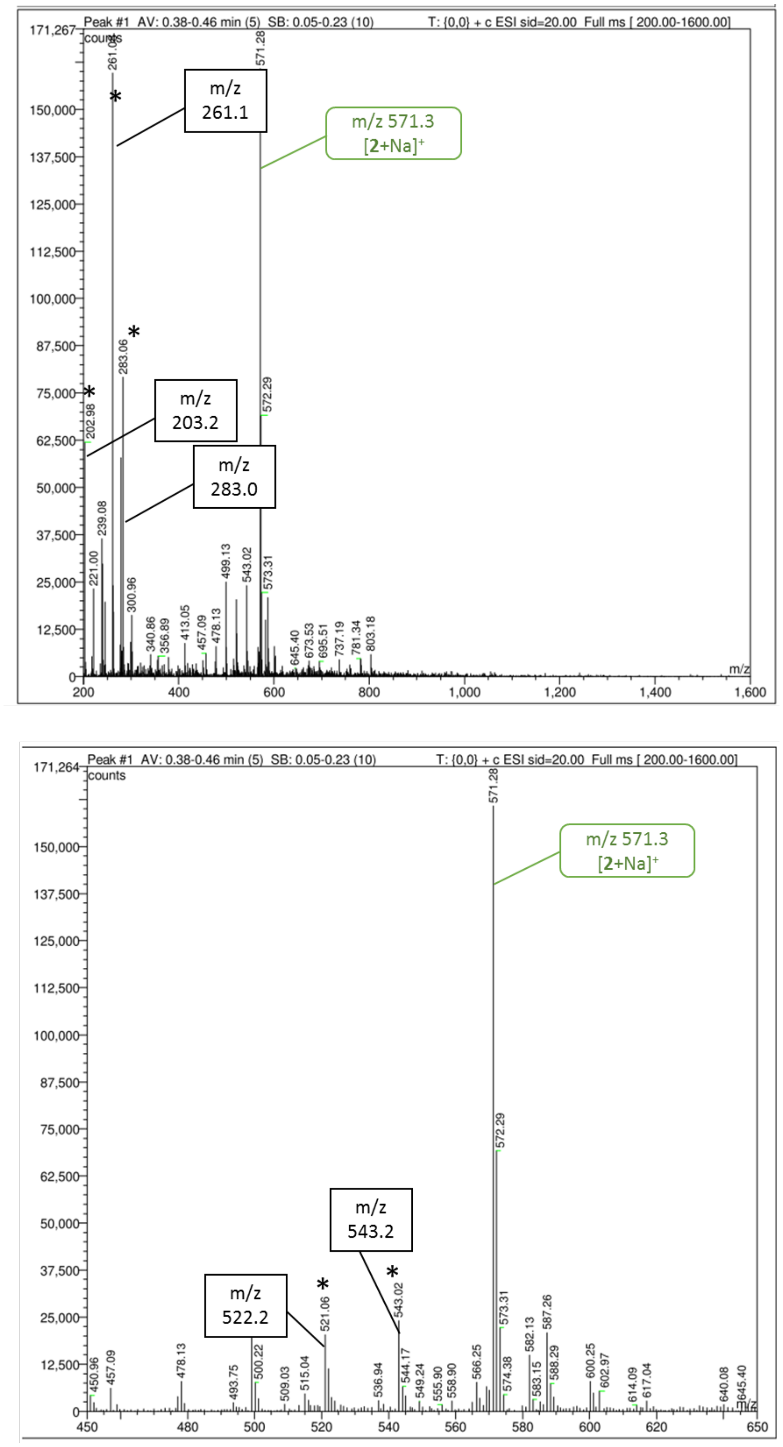

Figure S4C. ESI-MS spectra of [**3**]$(PF_6)_2$ (25 μM) in Opti-MEM medium (2.5% FCS, Pen/Strep, GlutaMax) after blue light irradiation (20 min, 12.6 $J.cm^{-2}$): full range (top) and zoom (bottom). Asterisks indicate the medium-characteristic peaks. The peak at m/z = 571.3 stands for the thioether-cholesterol ligand complexed to Na^+, *i.e.* [**2**+Na]$^+$ (calc. m/z = 571.4). No significant peak was observed for the unreacted compound [**3**]$^{2+}$ at m/z = 519.6.

6. Photo Cytotoxicity

Table S1. EC_{50} values for [**3**]$(PF_6)_2$ and CDDP (cisplatin), calculated from the logarithmic dose-response curve for two selected cell lines A431 (skin) and A549 (lung) after incubation for 6 h or 24 h respectively, followed by irradiation for zero or 10 min with blue light (454 nm, 6.3 J.cm^{-2}). The values are given in µM with confidence interval (95%).

Compound	t_{inc} (h)	t_{irr} (min)	EC_{50} (in µM) for A431 cells	for A549 cells
[**3**]$(PF_6)_2$	6	0	9.5 ± 1.1	5.9 ± 0.8
		10	9.6 ± 2.2	5.1 ± 1.1
	24	0	6.1 ± 0.8	5.1 ± 0.5
		10	3.2 ± 0.2	3.0 ± 0.2
CDDP	24	0	3.6 ± 0.6	4.3 ± 0.7
		10	4.4 ± 1.2	4.1 ± 1.3

7. Growing curves of A549 cancer cell line in presence of different compounds

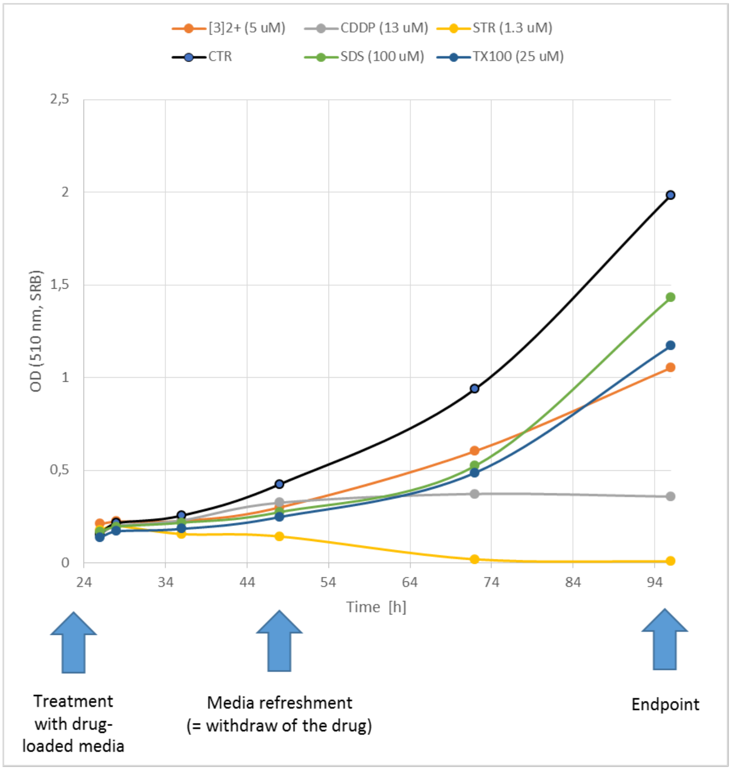

Figure S5: Evolution of the population of lung cancer cells A549 treated with medium only, [**3**]$(PF_6)_2$, CDDP (cisplatin), sodium dodecyl sulfate (SDS), staurosporine (STR), Triton-X (TX100), at different time points. After each time point the cells were fixated with ice-cold trichloracetic acid (10%), stained according to the SRB protocol (see Experimental Section, par. 8 in the main text), and the absorbance at 510 nm was measured.

8. MALDI-MS analysis with cells

Table S2: Significant cell and ruthenium specific peaks in the obtained MALDI-TOF spectra. A specific assignment is given as far as possible. For the cell specific peaks an indication of the effect of blue light irradiation is given. The pictogram "↑ " indicates a noticeable increase, "↓" a decrease, and "=" no changes of the signal intensity compared to the non-irradiated sample. The numbers of symbols should indicate the intensity of the trend, whereby e.g. ↑↑↑ stands for a change more than 3x, ↑↑ around 2x, and ↑ for at least more than one digit.

m/z	General assignment	Specific assignment	Effect of Light
**283**	Cells		↑↑↑
**348**	Cells		↓
**490**	Ruthenium compound	$[Ru(tpy)(bpy\text{-}H)]^{+}$	
**504**	Cells		=
**526**	Ruthenium compound	$[Ru(tpy)(bpy\text{-}H)Cl]^{+}$	
**543**	Cells		↑
**578**	Cells		=
**582**	Cells		↑
**600**	Cells		↓
**615**	Cells		↑↑↑
**644**	Ruthenium compound	$[Ru(tpy)(bpy\text{-}H)DHB]^{+}$	
**667**	Cells		↓
**696**	Cells		↓
**724**	Cells		↓
**755**	Cells		↓↓
**783**	Cells	Phosphocholin 36:4[1]	↓↓↓
**799**	Cells		↓
**809**	Cells		↓

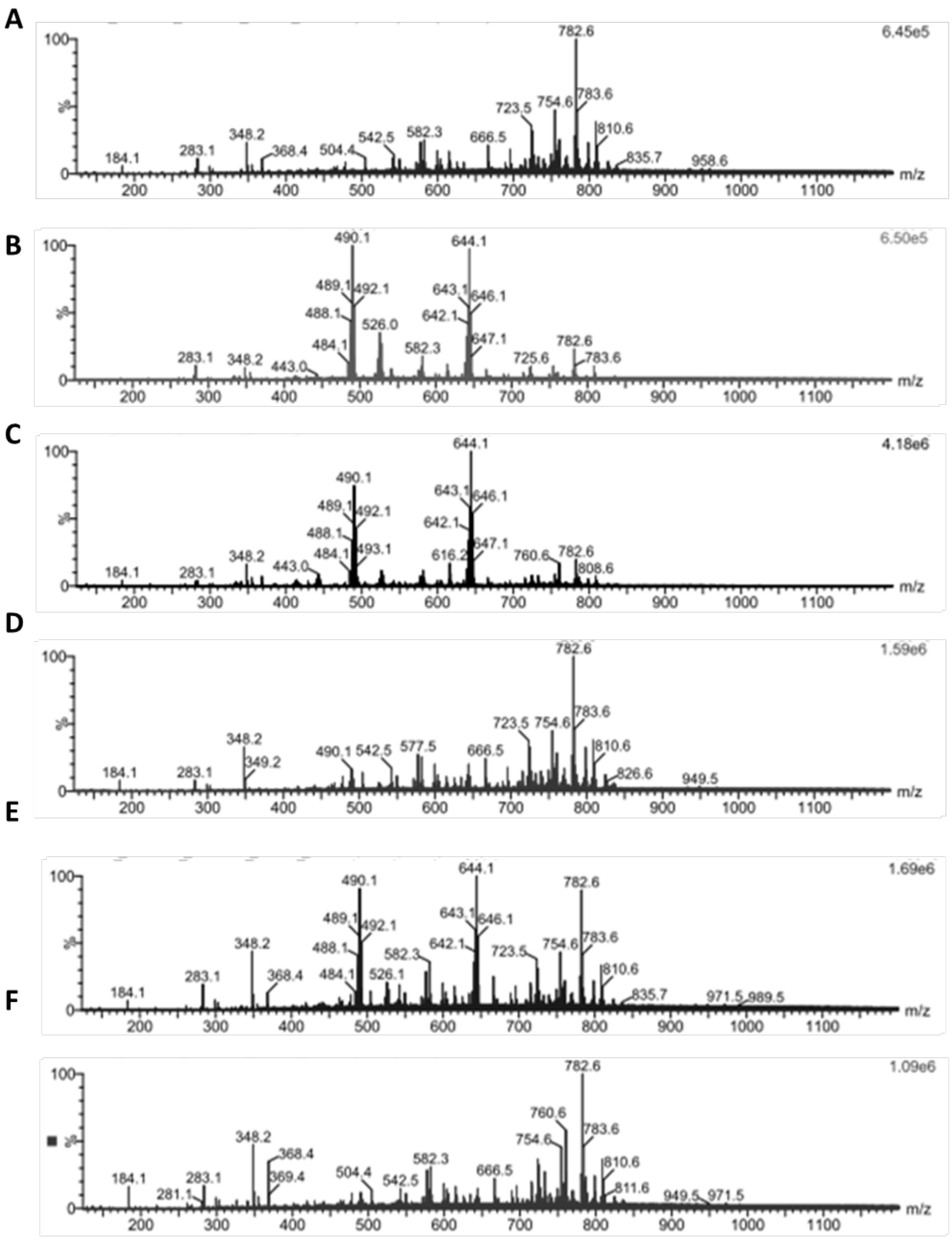

Figure S6. Representative MALDI-MS spectra of human lung cancer cells A549 treated in the dark with (**A**) medium only, (**B**) [**3**](PF_6)$_2$ (5 μM) for 24 h (**B**), [**3**](PF_6)$_2$ (1 μM) for 24 h (**C**), with [**1**](PF_6)$_2$ (50 μM) for 6 h (**D**), with [**3**](PF_6)$_2$ (5 μM) for 6 h (**E**), and with [**3**](PF_6)$_2$ (5 μM) for 1 min (**F**) (= negative control).

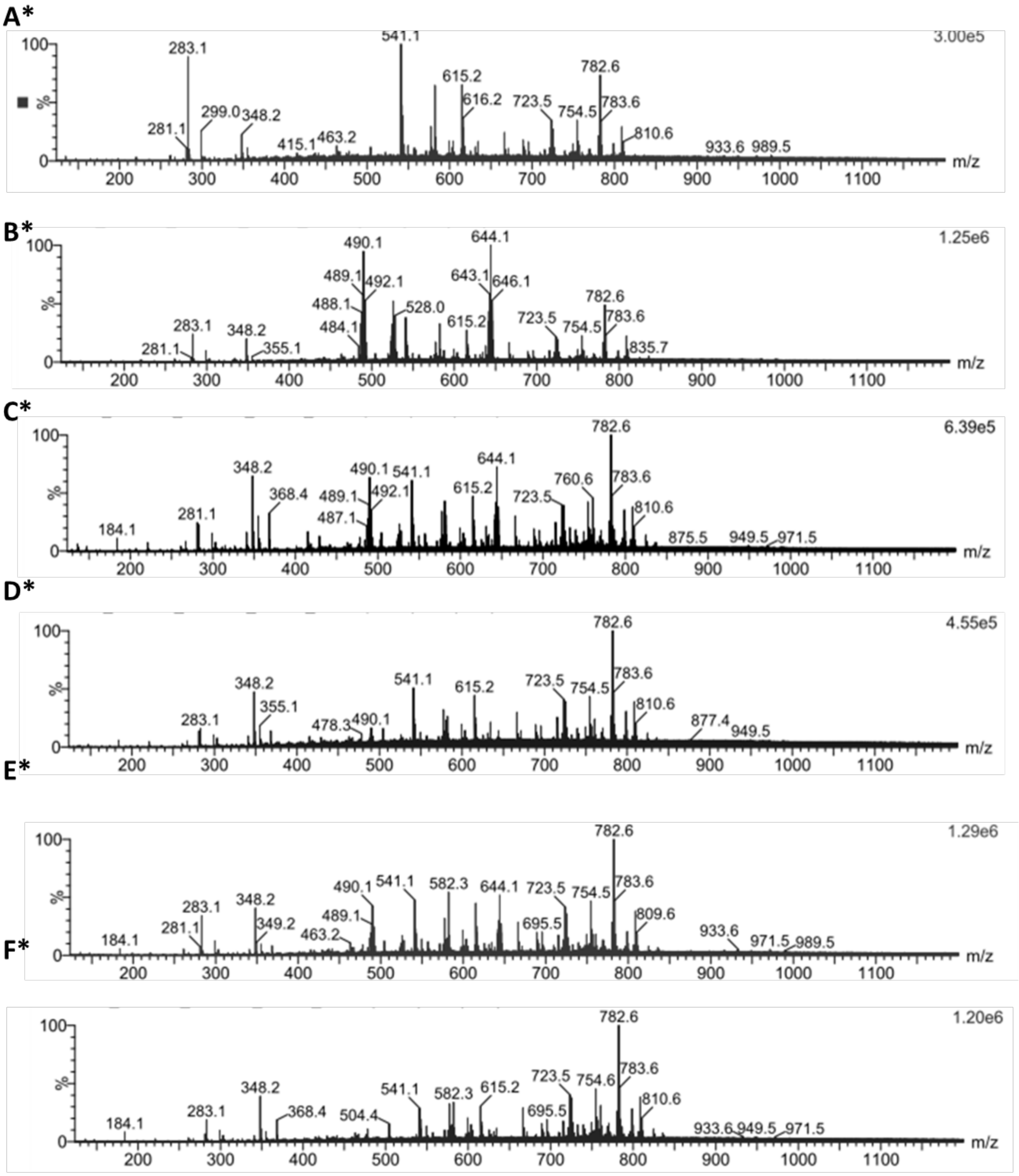

Figure S7. Representative MALDI-MS spectra of light-treated cells of the human lung cancer cell line A549 with medium only (**A***), [**3**]$(PF_6)_2$ (5 µM) for 24 h (**B***), [**3**]$(PF_6)_2$ (1 µM) for 24 h (**C***), with [**1**]$(PF_6)_2$ (50 µM) for 6 h (**D***), with [**3**]$(PF_6)_2$ (5 µM) for 6 h (**E***), and with [**3**]$(PF_6)_2$ (5 µM) for 1 min (**F***) (= negative control). Asterisks indicate measurements after irradiation with blue light (455 nm, 10 min, 6.3 J/cm²).

9. Microscopic investigation of living cells in the presence of surfactants in the dark

Surfactants are able to extract lipid-proteins and are frequently used to study extracted lipid-proteins. One method of protein reconstitution utilizes detergent micelles.[2,3] Most commonly are used detergents of nonionic character or ionic character. Ionic detergents generally lead to denaturing of the proteins in some extent. Denatured proteins often precipitate as they usually lose their water solubility. We investigated the effect of different detergents to compare the induced alteration of cell morphology with this seen after treatment with solutions of [**3**]$(PF_6)_2$. Here we used a representative of each detergent class, *i.e.*, Sodium dodecyl sulfate (SDS, a negatively charged detergent), Triton-X (a neutral detergent), and Cetyltrimethylammoniumbromide (CTAB a positively charged detergent). Figure S8 shows optical micrographs of A549 cells treated for 24 h with each of these detergents, for comparison with [**3**]$(PF_6)_2$ and with a control treated with medium only.

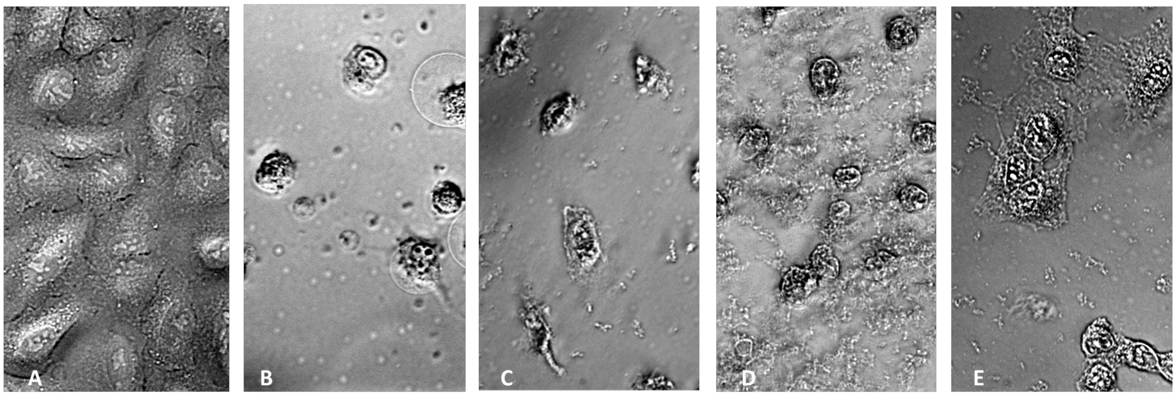

Figure S8. Micrographs (40x) of cells from the lung cancer cell line A549 after 24 h incubation with (A) medium only, (B) Triton X 100 (250 μM), (C) SDS (500 μM), (D) [**3**]$(PF_6)_2$ (12.5 μM), and (E) CTAB (10 μM).

10. DNA-Laddering Experiment

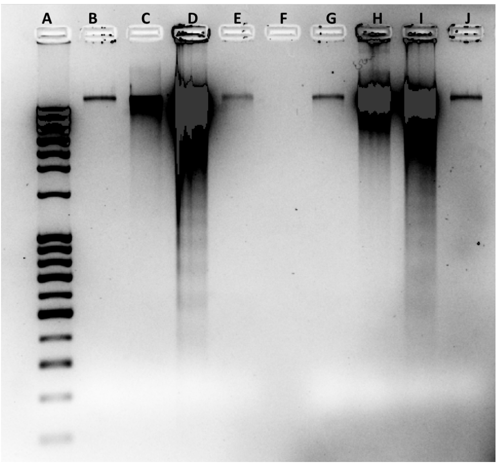

Figure S9: Gel electrophoresis of the DNA-laddering experiment. On an agarose gel (2%) were loaded the standard 1 kbp Plus DNA ladder (**A**), and the DNA extract of A549 cells incubated for 24 h with medium only (**B**), with CDDP (10 μM) (**C**), with Staurosporine (1 μM) (**D**), with [**3**]$(PF_6)_2$ (10 μM) (**E**). In the same order the ladders are given for 48 h incubation time (G to J).

11. Cell Cycle Investigation by Flow Cytometry

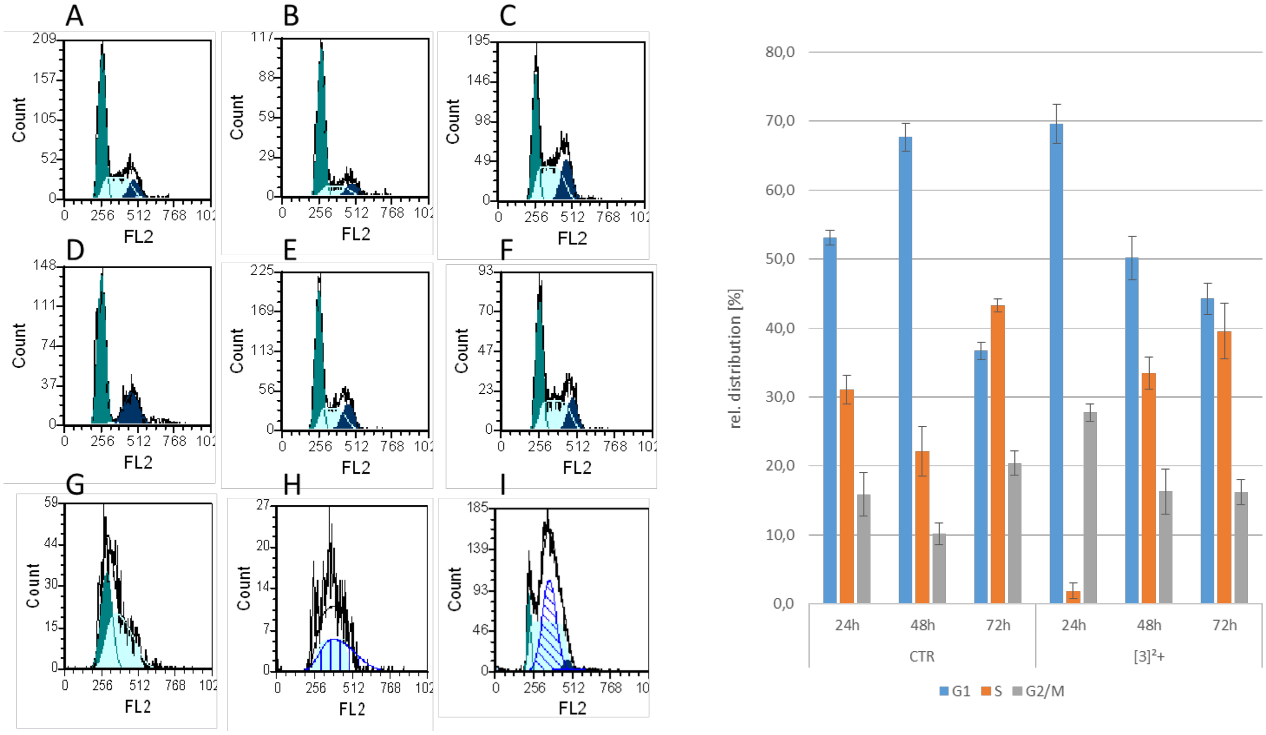

Figure S10. Representative flow cytometry histograms from the cell cycle investigation of living cells of a lung cancer cell line (A549) treated with [**3**]$(PF_6)_2$ in the dark, and drug-free control. Graphs A, B, and C represent the control cell population (no drug) after 24, 48, and 72 h, respectively. In the 2nd row the histograms obtained after treatment with [**3**]$(PF_6)_2$ (10 µM) are given for 24 h (**D**), 48 h (**E**), and 72 h (**F**). The bottom row shows the histograms obtained after treatment with CDDP (10 µM; positive control) for 24 h (**G**), 48 h (**H**), and 72 h (**I**). The bar graph on the right shows the relative distribution of the different growth phases of [**3**]$(PF_6)_2$ treated cells in comparison to the drug-free control (CTR), with standard errors.

12. Dye-exclusion assay (trypan blue)

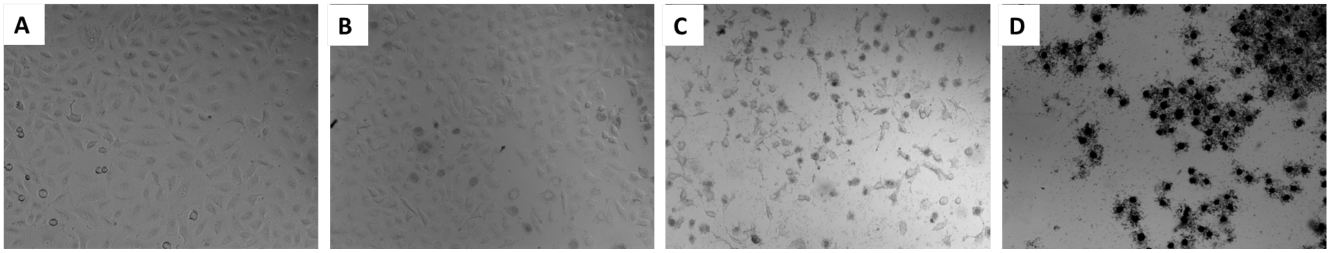

Figure S11. Representative micrographs (10x) of A549 cells treated with medium only (A), with 5 μM (B), 13 μM (C), and 25 μM [**3**](D) of $(PF_6)_2$.

13. Annexin V-Propidium Iodid based Apoptosis assay.

200 × 10^3 A549 cells were seeded in 35 mm petri dishes in Opti-MEM complete media (2 mL). After 24 h, the media was replaced with [**3**]Cl loaded media (10 μM, 2 ml), and incubated for 24 h. Thereafter the media was refreshed, and after additional 24 h all cells were collected, washed twice with PBS, and suspended in Annexin V binding buffer[4] (1 × 10^6 cells/mL). The cell suspension (100 μL) was stained with propidium iodide (5 μL, 10 μg/ml) and the Annexin V-FITC conjugate (3 μL, Bio connect) in the dark for 15 minutes. After addition of Annexin V binding buffer (200 μL) the cells were submitted to FACS measurement.[5] Quantification of the induced cell death was performed with FlowJo, using a standard protocol (Figure S12).[6]

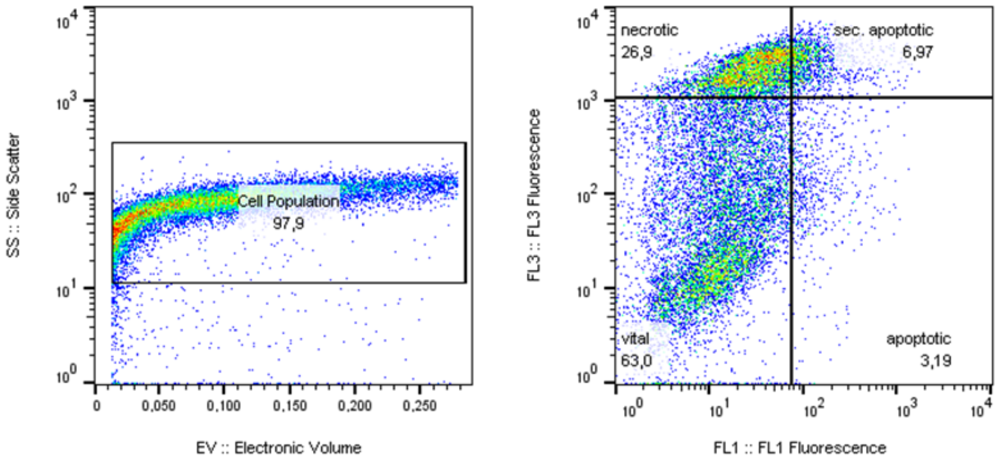

Figure S12. Representative flow cytometric density plots. Left: A selected to-be investigated cell population in the respective electronic volume vs SSC plot. Right: Annexin V - FITC (FL1, 525 nm) / propidium iodide (FL3, 670 nm) of A549 cells treated with [**3**]$(PF_6)_2$ (10 μM, 24 h drug incubation, 48 h total incubation). Majority of the dead cells hold a necrotic hallmark. Negligible apoptotic cells, indicated that apoptosis is not relevant.

14. Determination of the critical aggregate concentration (CAC)

The critical aggregate concentration (CAC) of a chemical compound is defined as the concentration at which further addition of amphiphilic molecules does not change the monomer concentration. Above this concentration the monomer molecules are in equilibrium with supramolecular aggregates of finite size. When these aggregates are micelles, the CAC is called critical micelle concentration (CMC). Here the formation of spherical aggregates is clear by transmission electron microscopy, however, as the type of aggregation (e.g. liposomes vs micelles) was not studied in detail, we report CAC in this article.

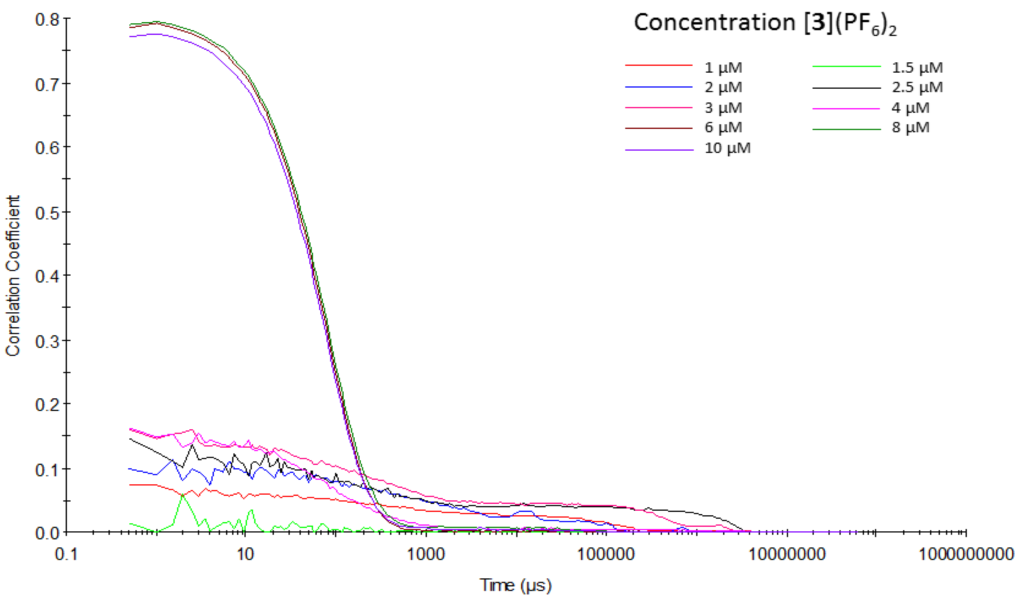

Figure S13. Representative correlation functions obtained by DLS for several concentrations of [**3**](PF_6)$_2$ in water. At a concentration ≥ 6 µM significant aggregate formation was observed, and some aggregation is already observed at the 3 µM level .

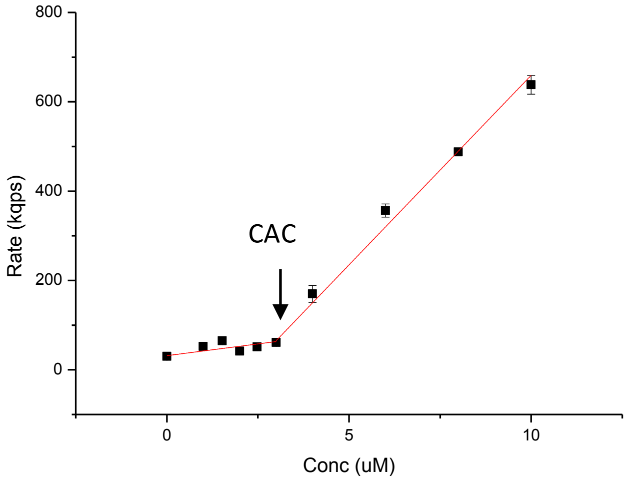

Figure S14. Representative plot of the intensity of scattered light (in kilocounts per second) obtained by DLS for samples containing various concentrations of [**3**](PF_6)$_2$ in deionized water. Bars represent errors of technical triplicate experiments. The intersection corresponds to the critical aggregate concentration (CAC). The average of two independent experiments was 3.5 ±0.5 µM.

15. Dose-response-curves of [3]$(PF_6)_2$ and CDDP treated cells and dependence on seeded cell numbers

A549 cell were seeded, treated, and processed in analogy to the dark cytotoxicity assay, but with different numbers of cells per well to investigate the effect of cell lipids versus compound [3]$(PF_6)_2$ (left) and CDDP (right). A clear difference was found for [**3**]$(PF_6)_2$, whereas CDDP showed very similar dose-response curves, independent of the starting numbers of cells.

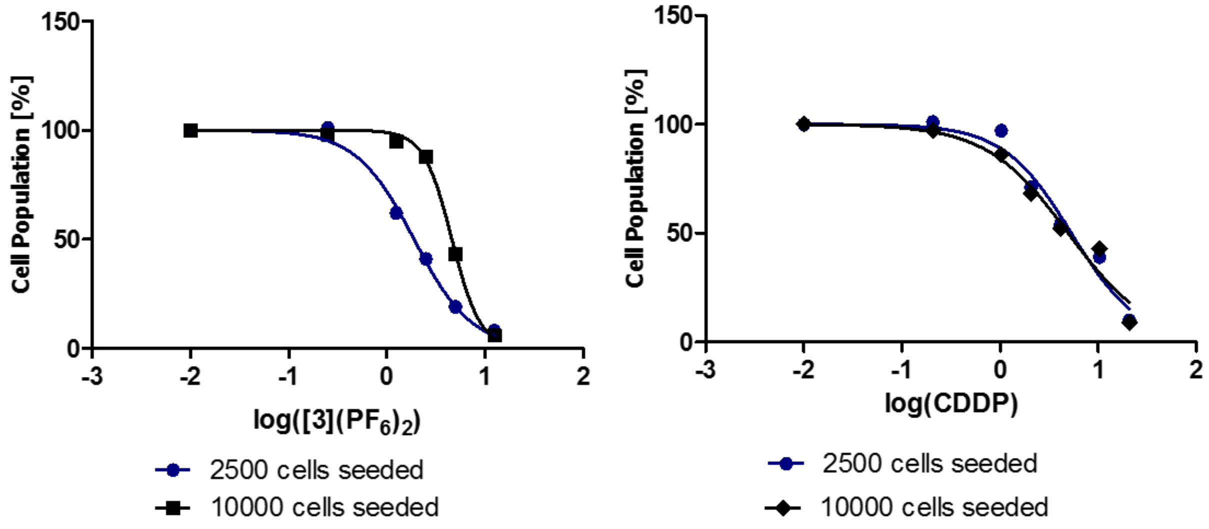

Figure S15. Dose-response curve of A549 cells treated with [**3**]$(PF_6)_2$ or CDDP. Concentration ranges did not change, but the number of cells seeded on day 1 was modified.

16. References

(1) Sparvero, L. J.; Amoscato, A. A.; Dixon, C. E.; Long, J. B.; Kochanek, P. M.; Pitt, B. R.; Bayır, H.; Kagan, V. E. *Chemistry and Physics of Lipids* **2012**, *165*, 545.

(2) Seddon, A. M.; Curnow, P.; Booth, P. J. *Biochimica et Biophysica Acta (BBA) - Biomembranes* **2004**, *1666*, 105.

3) le Maire, M.; Champeil, P.; Møller, J. V. *Biochimica et Biophysica Acta (BBA) - Biomembranes* **2000**, *1508*, 86.

(4) Koopman, G.; Reutelingsperger, C.; Kuijten, G.; Keehnen, R.; Pals, S.; van Oers, M. *Blood* **1994**, *84*, 1415.

(5) Koopman, G.; Reutelingsperger, C. P.; Kuijten, G. A.; Keehnen, R. M.; Pals, S. T.; van Oers, M. H. *Blood* **1994**, *84*, 1415.

(6) Williams, O. In *Apoptosis Methods and Protocols*; Brady, H. M., Ed.; Humana Press: 2004; Vol. 282, p 31.

(7) Israelachvili, J. N. *Intermolecular and Surface Forces: Revised Third Edition*; Elsevier Science, 2011.
